# Inverse Vulcanization of a Natural Monoene with Sulfur as Sustainable Electrochemically Active Materials for Lithium-Sulfur Batteries

**DOI:** 10.3390/molecules26227039

**Published:** 2021-11-22

**Authors:** Jian Xiao, Zhicong Liu, Wangnian Zhang, Ning Deng, Jijun Liu, Fulai Zhao

**Affiliations:** 1 School of Physics and Electronic Information, Shangrao Normal University, Shangrao 334001, China; LZC13177970359@163.com; 2 School of Materials Science and Engineering, Tianjin University, Tianjin 300072, China; 3 School of Materials Science and Engineering, Jiujiang University, Jiujiang 332005, China; zwn2003@126.com (W.Z.); dengning140127@126.com (N.D.); 4 School of Chemical and Environmental Sciences, Shangrao Normal University, Shangrao 334001, China; liujijun71@163.com

**Keywords:** poly(S-MVT), inverse vulcanization, sustainable, protonated thiazole nitrogen, cathode active material

## Abstract

A novel soluble copolymer poly(S-MVT) was synthesized using a relatively quick one-pot solvent-free method, inverse vulcanization. Both of the two raw materials are sustainable, i.e., elemental sulfur is a by-product of the petroleum industry and 4-Methyl-5-vinylthiazole (MVT) is a natural monoene compound. The microstructure of poly(S-MVT) was characterized by FT-IR, ^1^H NMR, XPS spectroscopy, XRD, DSC SEM, and TEM. Test results indicated that the copolymers possess protonated thiazole nitrogen atoms, meso/macroporous structure, and solubility in tetrahydrofuran and chloroform. Moreover, the improved electronic properties of poly(S-MVT) relative to elemental sulfur have also been investigated by density functional theory (DFT) calculations. The copolymers are utilized successfully as the cathode active material in Li-S batteries. Upon employment, the copolymer with 15% MVT content provided good cycling stability at a capacity of ∼514 mA h g^−1^ (based on the mass of copolymer) and high Coulombic efficiencies (∼100%) over 100 cycles, as well as great rate performance.

## 1. Introduction

Sulfur-containing polymers have been paid much more attention by scientists for almost a century due to their invaluable properties such as optical [1,2] and electrical [3] properties, self-healing properties [4], electrochemical properties [5,6,7,8], etc. They have been applied in high refractive index polymeric optics, stimuli-responsive polymeric systems, photocatalysis and semiconducting particles, and lithium–sulfur batteries [9]. 

In recent years, to meet the growing demand for energy consumption in the current society, more renewable energy sources are needed to supplement or replace the use of petroleum-based energy [10,11,12]. However, renewable energy in nature, such as solar energy, wind energy, tidal energy, etc., is discontinuous, so energy storage devices are needed to store energy for continuous use as needed. Lithium-sulfur (Li-S) batteries are considered to be one of the best choices for the next generation of energy storage systems because of their high theoretical energy density and natural abundance of sulfur [13,14,15]. As a result of the current technical level, however, there is still a long way to go for Li-S batteries in commercial applications because of some problems, such as sulfur not being conductive, intermediate lithium polysulfide generated during the sulfur cathode electrochemical reaction is dissolved in the electrolyte, and the volume expansion of cathode material happening in the reaction process. All of these seriously affect the life and safety of the whole battery. The use of sulfur-containing polymers as cathode materials has been an effective way to solve these problems, because polymers have various molecular structures and definable functional groups, and suitable monomer design enables the target polymer to have good properties such as ionic and electronic conductivity, high sulfur content, suitable viscosity, processability, and controllable morphology. These features all contribute to the performance of lithium-sulfur batteries, and the active sulfur is restrained in the polymer chain network through either chemical bonding or physical confinement, which can impede the dissolution of polysulfides in the electrolyte. Moreover, polymers can render an elastic framework which can hold about 20% volume expansion of sulfur. 

To prepare a new sulfur-containing polymer, in 2013, Pyun et al. pioneered inverse vulcanization synthesis of sulfur copolymer using elemental sulfur and 1,3-diisopropenylbenzene (DIB) as raw materials. This sulfur copolymer can accommodate a large percentage of sulfur—up to 99%—and presents excellent electrochemical performance compared to elemental sulfur when used as cathode active materials for Li–S batteries. Then, a number of sulfur copolymers are prepared by this facile and inexpensive method, such as poly(S-DVB) [16], poly(S-co-DiPhDY) [17], poly(S-r-BUMB18C6) [18], poly(S-BMI) [19], poly(OLA-r-S) [20], poly(S-r-Sty) [21], poly(S-r-UDOL) [22]. However, most of the raw materials of these polymers are petroleum products and are not renewable. In order to achieve sustainable development, it is necessary to develop new sulfur-containing polymers with sustainable materials as raw materials [23,24].

In this work, 4-Methyl-5-vinylthiazole (MVT), a nitrogen and sulfur-containing heterocyclic monoene compound, and natural product found in cocoa, *Passiflora edulia* Sims, etc., was first used to synthesize a novel sulfur copolymer poly(S-MVT) by inverse vulcanization with elemental sulfur. The as-synthesized polymers possess protonated thiazole nitrogen atoms, meso/macroporous structure, and solubility in tetrahydrofuran and chloroform. The electrochemical properties of the poly(S-MVT)s were investigated in detail. When used as cathode material of a Li–S battery, poly(S-MVT) (15% MVT) delivered an initial specific capacity of 872 mA h g^−1^ (based on the mass of poly(S-MVT)) with an initial coulombic efficiency of 96.57% and 514 mA h g^−1^ after 100 cycles at 0.1 C (1 C = 167.5 mA g^−1^). This provides a new choice for sustainable cathode materials for Li–S batteries.

## 2. Experimental Section

### 2.1. Sample Preparation

4-Methyl-5-vinylthiazole (98%) and elemental sulfur (99.95%) were purchased from Heowns and Aladdin, respectively, and used as received. The synthesis process of poly(S-MVT) was as follows. Elemental sulfur in a 20 mL rubber stopper sealed glass bottle equipped with a magnetic stirring bar was heated to 130 °C in a constant-temperature oil bath to form a clear yellow liquid. MVT was then added directly to the liquid sulfur via a syringe and the mixture was further heated to 185 °C for 10 min. Elemental sulfur and MVT were combined on a 3 g scale, in which MVT content was 10%, 15%, 20%, 30%, 40%, and 50% (mass percentage), respectively. The mixture was cooled to room temperature and the resulting product was scraped out of the bottle directly with a metal shovel.

### 2.2. Materials Characterization

^1^H NMR spectra were recorded on a Varian INOVA 500 MHz spectrometer using trimethylsilyl (TMS) as an internal standard with the solvent values (δ = 7.26 ppm for CDCl3-d1). Fourier transform infrared (FTIR) spectra were recorded on a FTIR 650 Spectrometer (BRUKER AXS GMBH) through KBr disks to analyze the chemical structure over a range of 400–4000 cm^−^^1^ at a resolution of 4 cm^−^^1^. Matrix-assisted laser desorption ionization time-of-flight mass spectroscopy (MALDI-TOF MS) spectra were measured with a Bruker Autoflex tof/tofIII mass spectrometer. X-ray diffraction (XRD) was performed on a D8 Advanced X-ray diffractometer (BRUKER AXS GMBH) with Cu Kα radiation (λ = 0.154 nm). The glass transition temperature (Tg) of the poly(S-MVT)s was determined using DSC (Q20, TA Instruments) with a heating rate of 10 °C min^−1^ from ambient temperature to 800 °C in N_2_ atmosphere. Differential scanning calorimetry (DSC) characterization was carried out using a TA Q20 instrument. X-ray photoelectron spectrum (XPS) measurement was performed on a PHI 1600 surface analysis system at a power of 450 W equipped with an Mg Kα anode. Morphology characterization of the materials was conducted by field emission scanning electron microscopy (FESEM) (Hitachi S-4800) and transmission electron microscopy (TEM) (JEOL JEM-2100F). The thermal stabilities of the copolymers were tested at a heating rate of 10 °C min^−^^1^ under N_2_ atmosphere, using a thermogravimetric analysis (TGA) system (NETZSCH STA 449C).

### 2.3. Computational Analysis

Density functional theory (DFT) calculations were carried out using Gaussian 09 software with the B3LYP hybrid exchange-correlation functional and 6-31G (d) basis set.

### 2.4. Measurement of Electrochemical Performances

The poly(S-MVT) powder was mixed with conductive carbon (Super P) and carboxymethylcellulose sodium salt (CMC) as a binder in a mass ratio of 8:1:1, respectively, and milled into a slurry with deionized water. The slurry was then cast onto a carbon-coated aluminum foil using a doctor blade and vacuum dried at 60 °C for 12 h. After drying, the foil was cut into disks with a diameter of 14 mm to fabricate the electrode. CR2032-type coin cells were assembled in a glovebox filled with an Ar atmosphere, using lithium metal as the counter electrode, Celgard 3500 membrane as the separator, and the solution of 0.38 M lithium bis(trifluoromethane)sulfonamide (LiTFSI) and 0.32 M lithium nitrate (LiNO_3_) in a 1:1 (*v*/*v*) mixture of 1,3-dioxolane and 1,2-dimethoxy ethane as the electrolyte. The cycling and rate performances were recorded on a Land battery measurement system (Wuhan, China) with a cut-off voltage of 1.5–3 V vs. Li/Li^+^. The specific capacity of the cell was calculated based on the poly(S-MVT) mass in the cathode. A cyclic voltammetry (CV) test was performed on an electrochemical workstation (CHI 660d) at a scan rate of 0.1 mV s^−1^ between 1.5 and 3 V. Electrochemical impedance spectroscopy (EIS) was conducted between 100 kHz and 0.01 Hz.

## 3. Results and Discussion

**Scheme 1:**
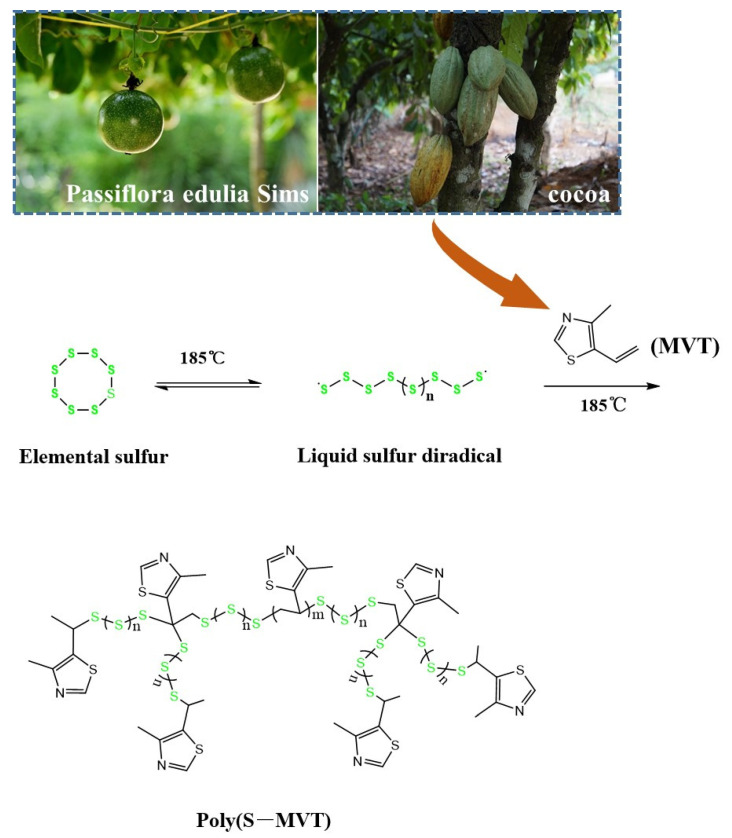
Synthesis of the Poly(S-MVT) and its proposed microstructure.

As the most basic and most abundant sulfur source, elemental sulfur is stable in thermodynamic stability under normal temperature. The elemental sulfur molecule is formed by eight sulfur atoms connected in a cyclo-S_8_, as shown in Scheme 1. On heating at 130 °C, the solid elemental sulfur melts into yellow liquid sulfur, therefore no additional solvent is needed in the inverse vulcanization synthesis process of poly(S-MVT)s. When heated over 159 °C, sulfur rings start to break, forming chain-like free radical monomers •S_8_•, which inevitably result in a reversible reaction of S_8_ ring-opening polymerization (ROP) into linear polysulfides with diradical chain ends, i.e., chain-like free radicals •(S_8_)_n_• with varying n values (*n* > 1). When the temperature reaches 185 °C, the sulfur chains are almost highly polymerized [25]. Owing to the existence of quantities of free radicals, the polymerization of vinyl monomers-MVT-can easily be initiated (Figure S1a). The sulfur diradicals can initiate the addition of vinyl double bonds of MVT, and they can also abstract vinyl hydrogen atoms from the MVT molecules to form vinyl radicals and sulfhydryl group. A vinyl radical then reacts with the sulfur diradical to form a C-S bond linked to a thiyl radical. After the rearrangement of radicals and then proton abstraction reaction, it is possible to form branched fragment and methyl group terminal (Figure S1a,b) [21]. These reactions occurred repeatedly to form a random poly(S-MVT) with branched fragments and methyl group terminals. 

Poly(S-MVT)s with MVT contents of 10%, 15%, 20%, 30%, 40%, 50% (mass percentage) were prepared. The color of the product turns from orange to red as the amount of MVT increases, and the color becomes darker and eventually becomes dark red (Figure S2). All the copolymers are soluble in organic solvents of chloroform, THF (Figure S3). Therefore, FTIR and liquid ^1^H NMR were first used to investigate the chemical structure of poly(S-MVT). 

**Figure 1:**
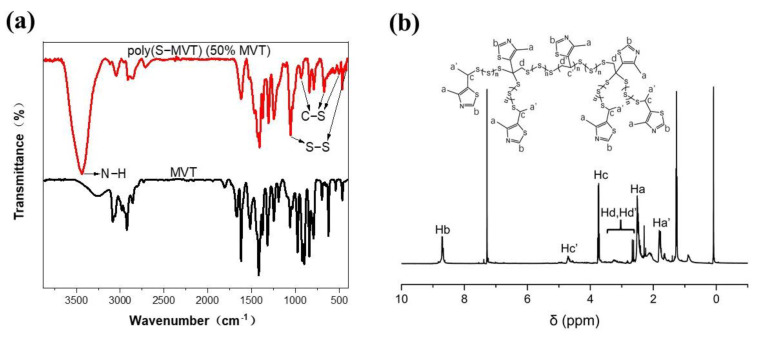
(**a**) FTIR spectra of MVT and Poly(S-MVT) (50% MVT); (**b**) HNMR spectra of poly(S-MVT) (15% MVT).

FTIR spectra of MVT and poly(S-MVT)s (Figure S4) indicated that the stretching vibration of C=C (1670 cm^−1^) and stretching (3086 cm^−1^) and out-of-plane bending vibrations (976 and 901 cm^−1^) of C-H arising from the vinyl group are absent in the spectrum of the Poly(S-MVT). Compared with MVT, poly(S-MVT) present new peaks, as clearly shown in Figure 1a. The peaks at 420 and 1055 cm^−1^ can be assigned to S-S bond, and the peaks at 505, 673, and 935 cm^−1^ may be assigned to the C−S bond between the vinyl group and sulfur chains. Moreover, a sharp peak appeared at 3437 cm^−1^, which can be attributed to the N-H stretching mode from the protonated nitrogen atoms of thiazole ring [26]. These results provide evidence for the successful polymerization reaction of elemental sulfur and MVT.

The ^1^H-NMR spectra of all poly(S-MVT)s (Figure S5) clearly indicated the complete conversion of MVT through disappearance of the signals corresponding to vinylic proton signals at 5.2, 5.4, and 6.7 ppm, regardless of the content of MVT in raw materials. As shown in Figure 1b, new signals appeared in the range between 5 and 1.5 ppm and were attributed to protons of methane (Hc, Hc’), methylene (Hd, Hd’), and methyl (Ha’) units from the structure that we proposed in Scheme 1. Thermogravimetric analysis (TGA) of poly(S-MVT)s showed first decomposition steps around 200 °C, which were very close to that of elemental sulfur [27,28,29], and can be ascribed to the volatilization of sulfur. The second steps around 350 °C were related to the weight loss of MVT (Figure S6). The dependence of the second step on the MVT content indicates that the feed ratio of MVT was completely conserved in the copolymers. 

**Figure 2:**
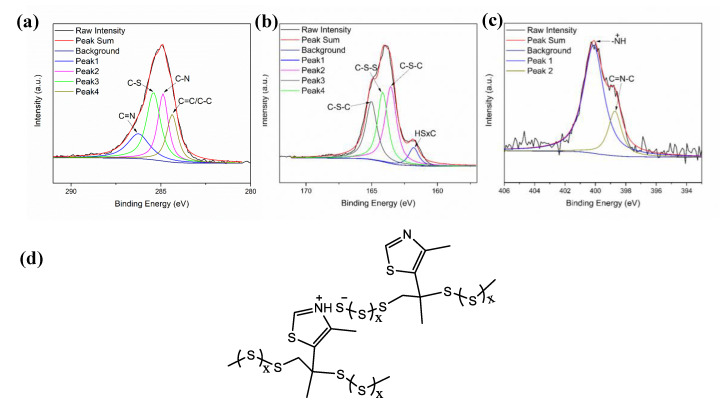
High-resolution (**a**) C1s, (**b**) S2p, and (**c**) N1s XPS spectra of poly(S-MVT) (15% MVT). The black curves are the experimental data, and the colored curves are deconvolution fits. (**d**) Proposed partial structure of protonated poly(S-MVT).

The XPS spectra in the C 1s region (Figure 2a) for poly(S-MVT) reveal that the C 1s band can be deconvoluted into four peaks at 284.4, 284.9, 285.4, and 286.3 eV, which could be attributed to C=C/C-C bonds, C-N bonds, C-S bonds, and C=N bonds, respectively. In the S 2p spectrum of the poly(S-MVT) (Figure 2b), the peaks at 161.8 eV and 164.2 eV are due to the HS_x_C [30] and C-S-S, respectively. The two peaks at 163.6 and 165.0 eV arise from the C-S-C [26]. This further confirmed the sulfur species have successfully linked to the thiazole molecules. The high-resolution N 1s XPS spectrum of poly(S-MVT) (Figure 2c) can be deconvoluted into two peaks at 398.7 and 400.1 eV, corresponding to C=N-C, and C-NH^+^ [26,31], respectively. The appearance of N 1 s peak at 400.1 eV further confirmed that some nitrogen atoms are protonated, i.e., the tertiary nitrogen atom of thiazole ring forms a salt with the H proton, and the H proton may be originated from the SH. This means that there may be a moiety as illustrated in Figure 2d existing in the poly(S-MVT). This structure could facilitate the electrochemical reaction rate when used as a cathode material [31,32,33].

**Figure 3:**
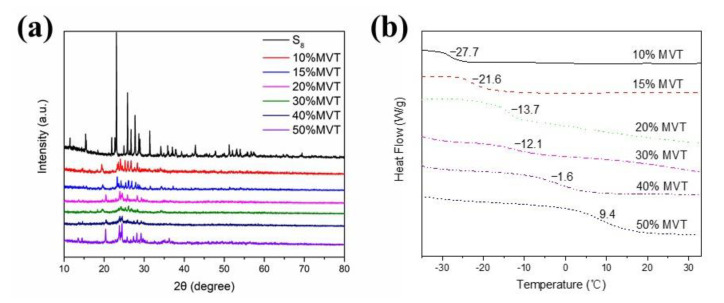
(**a**) XRD patterns of poly(S-MVT)s with different MVT content. (**b**) DSC thermograms of poly(S-MVT)s (second scan) with different MVT content.

XRD is one of the most efficient measurements to analyze elemental sulfur in the sulfur polymers based on S_8_. As Figure 3a shows, the transformation of crystalline state intuitively demonstrated the involvement in the polymer. Irrespective of the content of MVT, the diffraction peaks of crystal sulfur are extremely weak and even disappeared when the MVT content is over 15%. Moreover, new XRD peaks that are not present in the elemental sulfur appear in the poly(S-MVT)s, and the peak positions are concentrated in the range of 19.2–20.7° and 23.4–24.2°, demonstrating the elemental sulfur is almost fully polymerized in the polymer. Thermal analysis was also conducted on the poly(S-MVT) with varying MVT content to further confirm the formation of the copolymer. DSC revealed that poly(S-MVT)s with compositions that ranged from 10 to 50% MVT exhibited glass transition temperature (Tg) values from −27.7 °C to 9.4 °C, and the Tg values increase with MVT content progressively (Figure 3b). MALDI-TOF MS spectrum (Figure S7) showed that the molecular weight of the polymer was less than 1600 and concentrated in 568, which indicated that the low glass transition temperature and good solubility of the copolymers might be due to the low molecular weight. 

**Figure 4:**
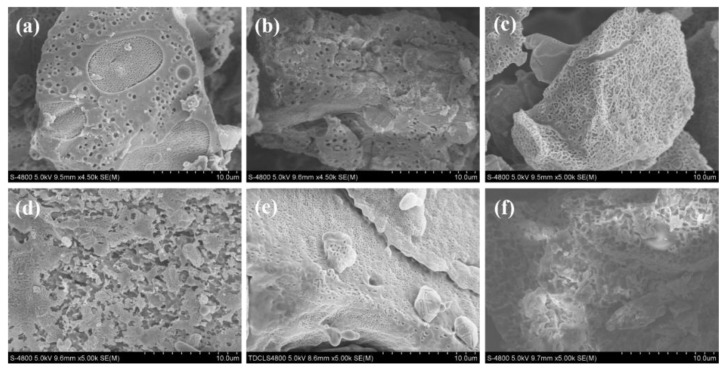
SEM images of poly(S-MVT)s with different MVT contents of (**a**) 50%, (**b**) 40%, (**c**) 30%, (**d**) 20%, (**e**) 15%, (**f**) 10%.

**Figure 5:**
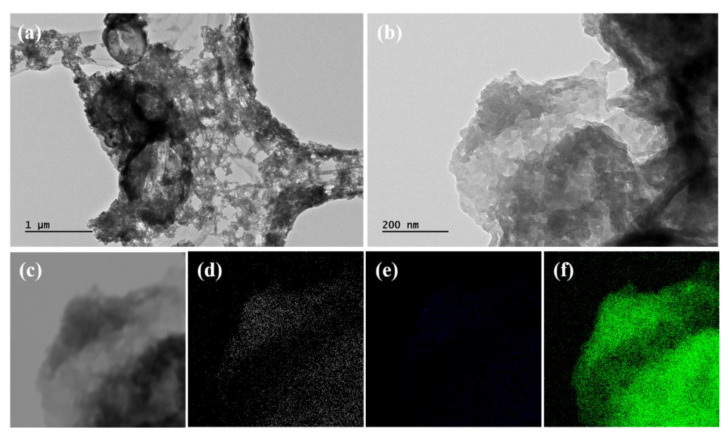
TEM images of poly(S-MVT) (15% MVT): (**a**) low magnification TEM image, (**b**) high magnification TEM image, (**c**) selected area image for mapping and corresponding elemental mappings of (**d**) carbon, (**e**) nitrogen, and (**f**) sulfur for poly(S-MVT) (15% MVT).

The morphologies and structures of the poly(S-MVT)s were examined by SEM and TEM measurements. As shown in Figure 4, all the poly(S-MVT)s had abundant pores on the external surface. This meso/macroporous structure was further confirmed by TEM (Figure 5a,b). The high TEM magnification image (Figure 5b) clearly showed that the pore diameters of poly(S-MVT) (15% MVT) were in the range of 30 to 300 nm. Furthermore, high-resolution elemental mapping analysis of a selected area (Figure 5c) indicates that C, N, and S elements are distributed uniformly throughout the entire poly(S-MVT) (Figure 5d–f). This merit is a benefit for enhanced fast ionic conduction and mass transportation when used in cathode of a Li-S battery. Moreover, the porous structure can help suppress the volume expansion of active sulfur during charging and discharging process [34,35]. The formation mechanism of the porous structure is not clear and needs further study.

**Figure 6:**
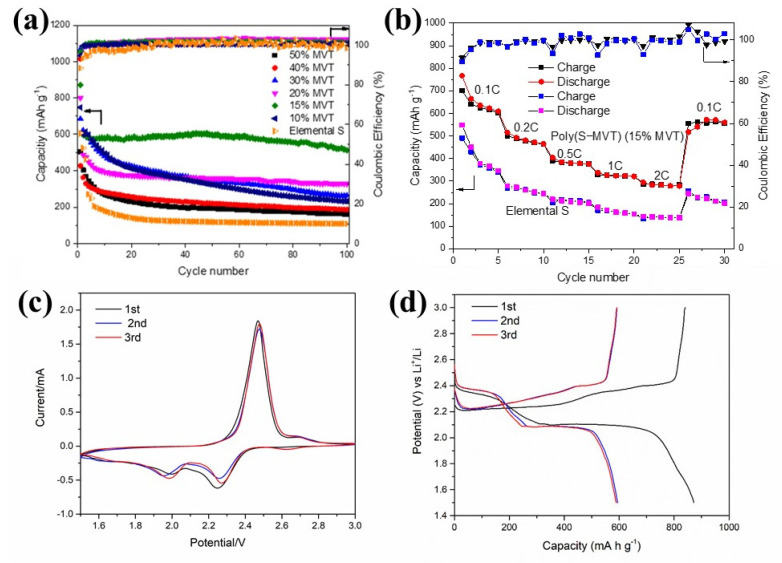
(**a**) Galvanostatic charge–discharge cycling of different poly(S-MVT)s at 0.1 C (capacity calculated based on the mass of poly(S-MVT)) and elemental sulfur (S). (**b**) Rate capacity of the poly(S-MVT) (15% MVT) copolymer and elemental sulfur S based electrodes at varied rates. (**c**) Cyclic voltammograms of the poly(S-MVT) (15% MVT) copolymer cathode at a scan rate of 0.1 mVs^−1^. (**d**) Charge–discharge profiles of Li-S batteries with poly(S-MVT) (15% MVT) copolymer cathode at 0.1 C.

The electrochemical properties of poly(S-MVT) were investigated in detail through Li-S cells assembled with these copolymer-based cathodes. As is exhibited in Figure 6a, for all the copolymers, the specific capacities of the batteries, which are higher than that of elemental sulfur, decline rapidly in the first few cycles, but then tend to be stable. Among the six copolymers, poly(S-MVT)s with MVT in 15–20% presented better cyclic performance. The poly(S-MVT) (15% MVT) showed the best performance which exhibited a high initial discharge capacity of 872 mAh·g^−1^ (based on the mass of poly(S-MVT)) and a reversible capacity of 597 mAh·g^−1^ at 2nd cycle at 0.1 C (1 C = 167.5 mA·g^−1^). After 100 cycles, the maintained capacity is up to 514 mAh·g^−1^, which is comparable and even superior to the recently reported sulfur-containing polymers (Table S1). This monomer content-dependent electrochemical performance change rule is similar to that of the previously reported sulfur copolymers [36]. To further investigate the electrochemical mechanism, a cyclic voltammetry (CV) test was conducted for the poly(S-MVT) (15% MVT)-based cathode. The CV result of poly(S-MVT)s presented similar electrochemical behavior to that of elemental sulfur. It can be seen from Figure 6c that at the first scan cycle, two reduction peaks appear at around 2.25 and 2.00 V, which can be related to the formation of higher order polysulfides and lower order sulfides, respectively. One oxidation peak at 2.47 V was observed, which can be attributed to the conversion of low-order sulfides to high-order polysulfides and eventually to sulfur. The oxidation peak at 2.47 V varies little throughout the three cycles and indicates the stability of electrochemical oxidation reactions. The well-maintained oxidation and reduction peaks in three cycles indicates that the electrochemical reaction is of good reversibility. Thus, the possible electrochemical reactions of the poly(S-MVT) based cathode are mainly polysulfide-based reactions, as is illustrated in Figure S8. Charge–discharge profiles further revealed the electrochemical reaction mechanism of the poly(S-MVT)-based Li-S batteries (Figure 6d). Two distinct plateaus were observed during the first discharge process, which correspond well to the CV profiles. The high plateau at 2.3 V was assigned to the formation of higher order polysulfides (C-S_x_-Li and Li_2_S_x_, x = 4–8), while the low plateau at 2.0 V was assigned to the formation of lower order sulfides (C-S_x_-Li and Li_2_S_x_, x = 1–3). 

**Figure 7:**
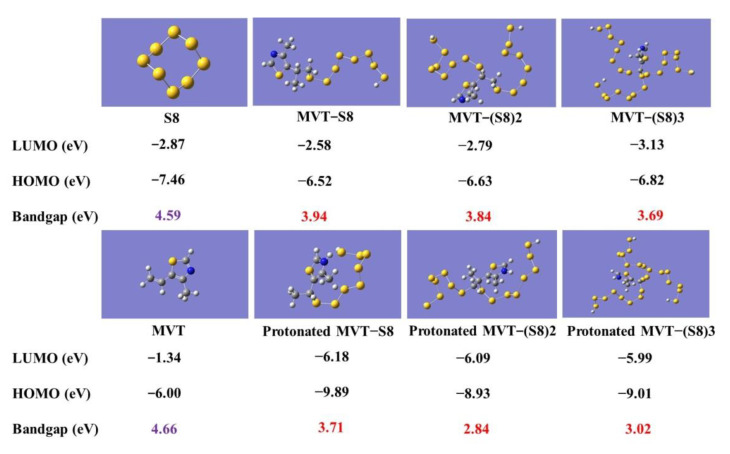
HOMO–LUMO bandgaps of elemental sulfur (S8), MVT, and poly(S-MVT)s with different polysulfide chains (-S8-). The band gap of MVT is 4.66 eV, which decreases greatly as a result of the polysulfide chain attachments.

**Figure 8:**
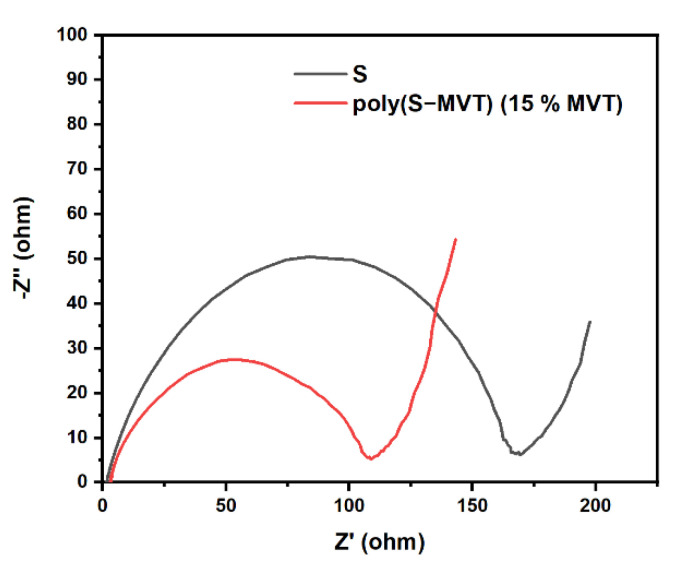
EIS spectra of the fresh S cathode and poly(S-MVT) (15% MVT) cathode.

It is worth noting that the initial coulombic efficiency of poly(S-MVT) (15% MVT) based Li-S cell is 96.57%, and after the second cycle, the coulombic efficiencies are all around 100%. Rate capability test showed that at C-rate of 0.1, 0.2, 0.5, 1, and 2, the poly(S-MVT) (15% MVT) delivered capacities of 609, 465, 376, 321, and 288 mAh·g^−1^ (Figure 6b), respectively, indicating a promising rate performance (far better than that of elemental sulfur). To further investigate the reasons for the excellent electrochemical performance of poly(S-MVT), we employed DFT calculations to study the HOMO–LUMO bandgap energy changes of poly(S-MVT). As Figure 7 shows, with the increase of polysulfide chains (-S8-) connected to MVT, the bandgap values decreased gradually. In addition, the protonated N on the MVT reduced the bandgap even more, which is much lower than that of elemental sulfur, thus making the poly(S-MVT)’s rate performance better [37,38]. This can be demonstrated by the electrochemical impedance spectroscopy (EIS) results. As Figure 8 shows, Nuquist curves of fresh S cathode and poly(S-MVT) (15% MVT) cathode consist of semicircles in the high frequency region and straight lines in the low frequency region. The semicircle diameter of the Nuquist curve represents the charge transfer resistance. It is obvious that the charge transfer resistance of poly(S-MVT) (15% MVT) cathode is much lower than that of S cathode, indicating better electronic conductivity and kinetics of the poly(S-MVT) (15% MVT). Hence, the stable cycle performance and excellent rate performance of poly(S-MVT) may be ascribed to the stable chemical structure linked by C-S bond, the protonated nitrogen atoms in thiazole ring, and the porous structure. The exceptional protonated structure, low bandgap, and promising electrochemical performance provides a new choice for the design of sustainable cathode materials for Li-S batteries.

## 4. Conclusions

In this work, we synthesized a novel soluble inverse vulcanization copolymer using elemental sulfur and a natural monoene 4-Methyl-5-vinylthiazole (MVT) in a fast one-pot solvent-free method. The poly(S-MVT)s were solublized well in chloroform and THF, and had a meso/macroporous structure, which is beneficial to constructing the ion electron transport channel when employed in Li-S battery cathode material. The unique thiazole ring structure brings the poly(S-MVT) protonated N after polymerization, which can facilitate the improvement of conductivity, and the binding and stabilization of polysulfide during cycling in the Li-S battery. All the copolymers with MVT from 50–90% are electrochemical active. Poly(S-MVT) (15% MVT) delivered an initial specific capacity of 872 mA h g^−1^ (based on copolymer) with initial coulombic efficiency of 96.57% and 514 mA h·g^−1^ after 100 cycles at 0.1 C (1 C = 167.5 mA g^−1^). The coulombic efficiencies are all around 100% after the second cycle. The unique merits of poly(S-MVT) make it easy to process and produce economical, environmentally friendly, sustainable, high-performance lithium-sulfur battery cathode materials, and can be used in many other applications.

## Data Availability

The data presented in this study are available on request from the corresponding author.

## Supplementary Materials

The following are available online, Figure S1: Proposed polymerization mechanism for poly(S-MVT) copolymer, Figure S2: Photographs of poly(S-MVT) with different MVT content, MVT content stated above, Figure S3: Photographs of poly(S-MVT)s in CS2, THF and chloroform, MVT content stated above, Figure S4: FT-IR spectra for MVT and all poly(S-MVT)s at different MVT content, MVT content stated above, Figure S5: HNMR spectra for MVT and all poly(S-MVT)s at different MVT content, MVT content stated above, Figure S6: TGA curves of poly(S-MVT)s with different MVT content, Figure S7: MALDI-TOF MS spectrum of poly(S-MVT) (50% MVT), Figure S8: The possible electrochemical reactions of the poly(S-MVT) based cathode, Table S1: Characteristics and electrode performance comparison of the present work with the recently reported literature.

## Sample Availability

Samples of the compounds (such as S-MVT) are available from the authors.
