# Supplementary material for: Inverse Vulcanization of a Natural Monoene with Sulfur as Sustainable Electrochemically Active Materials for Lithium-Sulfur Batteries
Source: Molecules. 2021 Nov 22;26(22):7039. doi: 10.3390/molecules26227039 (PMC8618051; doi:10.3390/molecules26227039)
Supplement: Supplementary file 1 [file molecules-26-07039-s001.zip › molecules-1451079-supplementary.pdf]

Supporting Information

Inverse Vulcanization of a Natural Monoene with Sulfur as Sustainable Electrochemically Active Materials for Lithium-Sulfur Batteries

Jian Xiao [a, b,*#], Zhicong Liu [a], Wangnian Zhang [c], Ning Deng [c], Jijun Liu [d] and Fulai Zhao[b,*#]

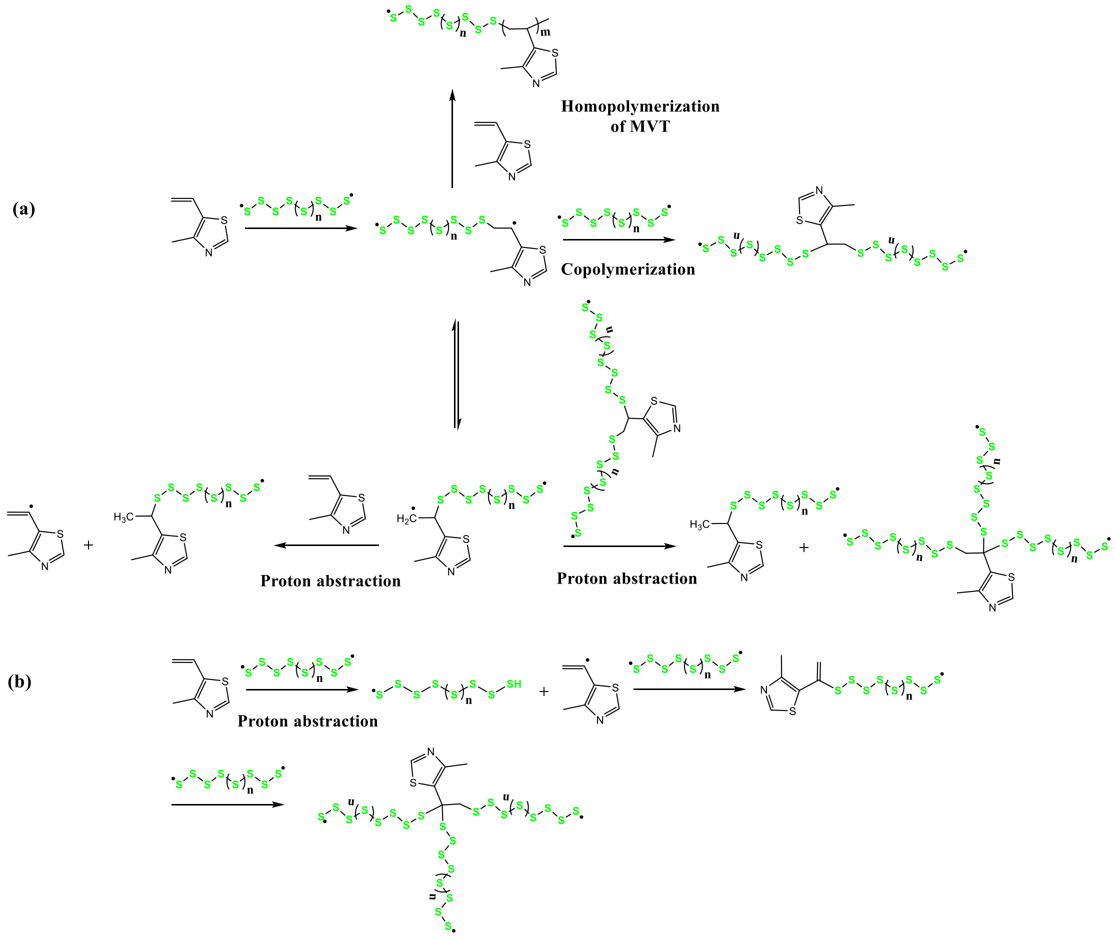

Figure S1 Proposed polymerization mechanism for poly(S-MVT) copolymer.

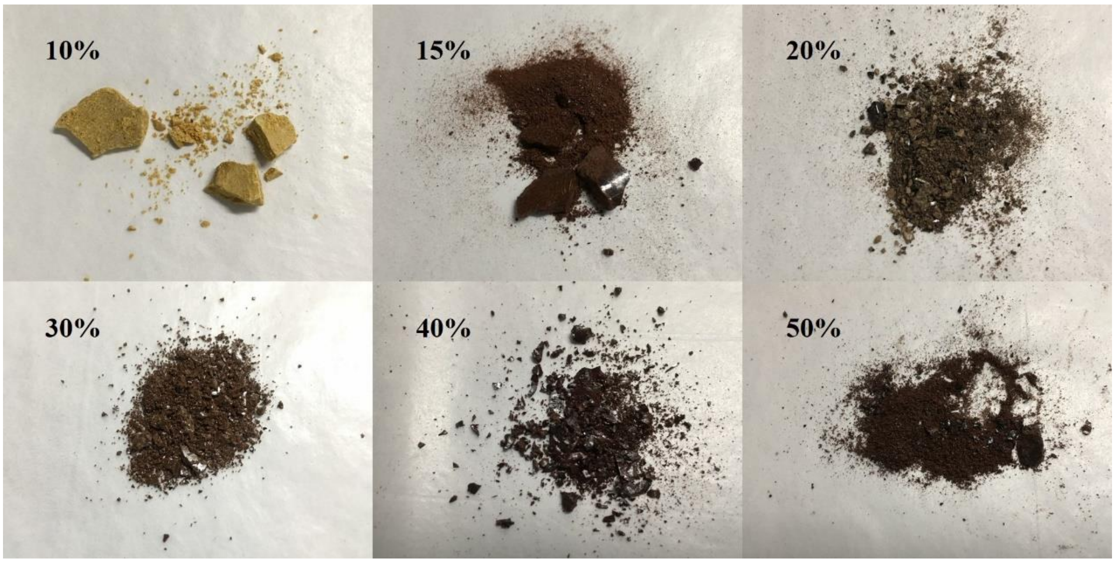

Figure S2 Photographs of poly(S-MVT) with different MVT content, MVT content stated above.

In order to test the solubility of the copolymer, we selected poly(S-MVT)s with MVT content of 10%, 30%, and 50% for testing. As shown in Figure S2, all the three poly(S-MVT)s have good solubility in THF and chloroform but can only partially dissolve in CS_2.

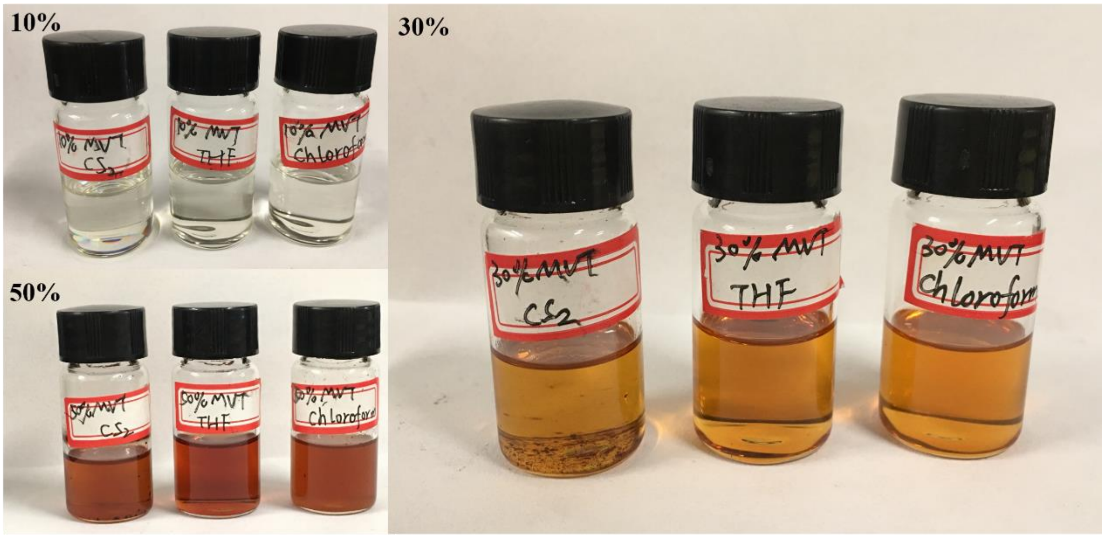

Figure S3 Photographs of poly(S-MVT)s in CS_2, THF and chloroform, MVT content stated above.

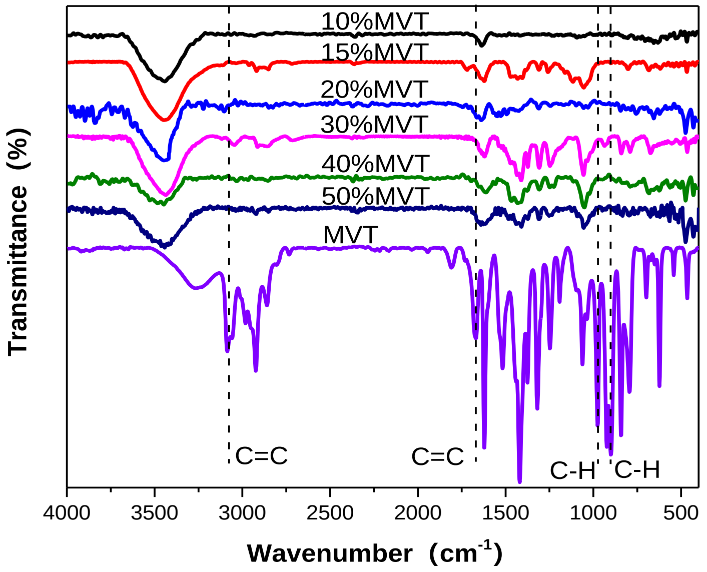

Figure S4 FT-IR spectra for MVT and all poly(S-MVT)s at different MVT content, MVT content stated above.

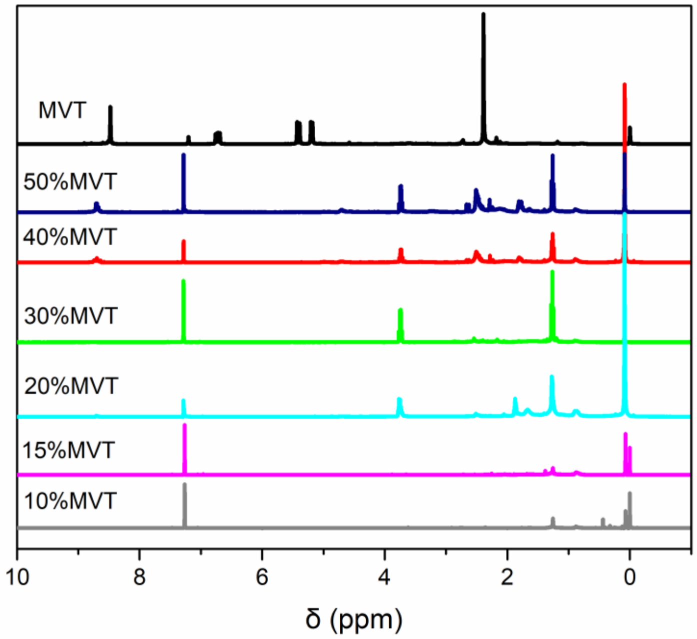

Figure S5 HNMR spectra for MVT and all poly(S-MVT)s at different MVT content, MVT content stated above.

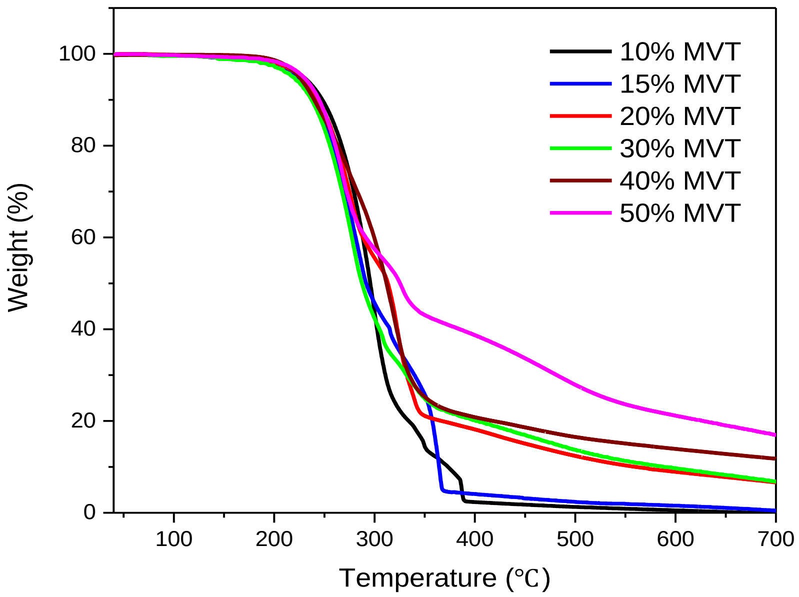

Figure S6 TGA curves of poly(S-MVT)s with different MVT content.

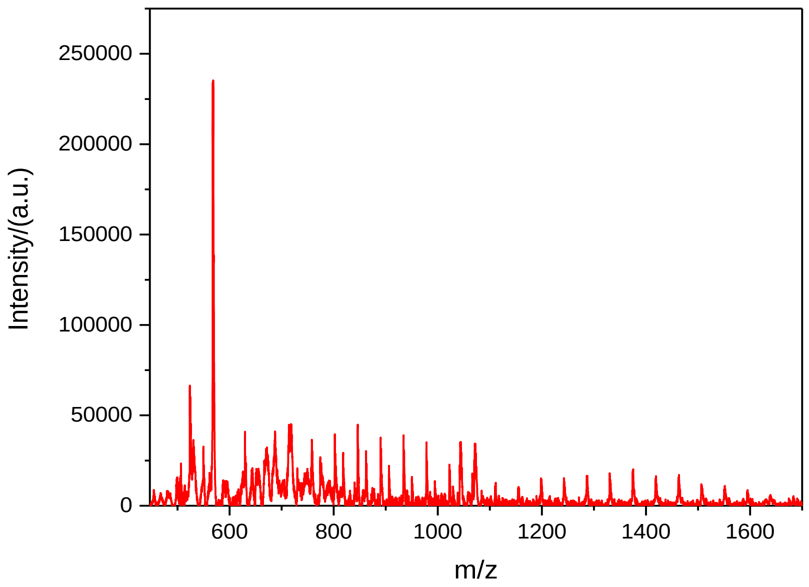

Figure S7 MALDI-TOF MS spectrum of poly(S-MVT) (50% MVT).

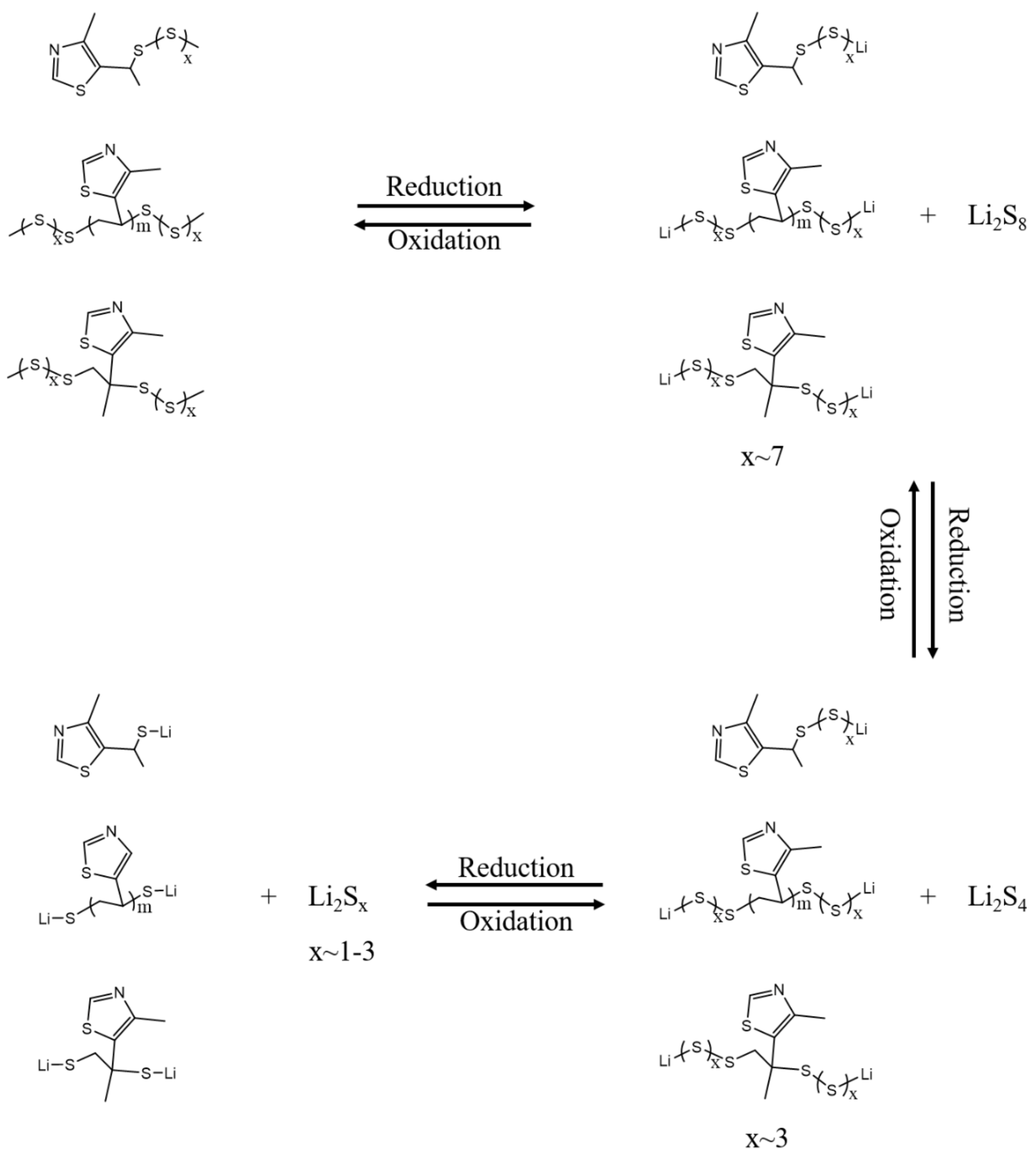

Figure S8 The possible electrochemical reactions of the poly(S-MVT) based cathode.

Table S1 Characteristics and electrode performance comparison of the present work with the recently reported literature.

Sulfur-containing polymers	Monomer	Renewable/ Sustainable	electrode performance	Reference
poly(S-co-EAE)	eugenol allyl ether	Yes	557 mAh g^{-1} based on S after 100 cycles at 0.1C	[1]
SVOCs	vegetable oil	Yes	554 mAh g^{-1} based on S after 100 cycles at 0.1C	[2]
Si-69 ($C_9H_{21}O_3SiS_4SiO_3H_{21}C_9$)	None	No	163 mAh g^{-1} even after 200 cycles at 0.2 Ag^{-1}	[3]
poly(S-Sty-P)	styrene-peanut oil	No	611 mAh g^{-1} based on S after 100 cycles at 0.2C	[4]
poly-(S-r-p)p	hexakis(styrene oxy)cyclotripho sphazene	No	350 mAh g^{-1} based on S after 100 cycles at 0.5C	[5]
poly(S-MVT)	4-Methyl-5-vinylthiazole	Yes	514 mAh g^{-1} based on copolymer (605 mAh g^{-1} based on S) after 100 cycles at 0.1C	This work

[1] A. Hoefling，T.N. Dan，Y.J. Lee，S.W. Song，P. Theato. A sulfur–eugenol allyl ether copolymer: a material synthesized via inverse vulcanization from renewable resources and its application in Li–S batteries. Mater. Chem. Front. 2017, 1,1818.

[2] Yamabuki, K., Itaoka, K., Shinchi, T., Yoshimoto, N., Ueno, K., Tsutsumi, H.. Soluble sulfur-based copolymers prepared from elemental sulfur and alkenyl alcohol as positive active material for lithium-sulfur batteries. Polymer 2017, 117: 225-230.

[3] Xing, C., Xue, P., Gu, X., Lai, C.. Communication—Organic Silane Coupling Agent Si-69: A New Organosulfur Cathode Material for Rechargeable Lithium Batteries. J. Electrochem. Soc. 2018, 165(16):A3782-A3784.

[4] Wang, J. and Zhang, S. A Novel Sulfur-Based Terpolymer Cathode Material for Lithium–Sulfur Battery. Energy Technol. 2020, 8(5), 2000057.

[5] Serkan Yeşilot, Sedat Küçükköylü, Emrah Demirb, Rezan Demir-Cakan. Phosphazene based star-branched polymeric cathode materials via inverse vulcanization of sulfur for lithium–sulfur batteries. Polym. Chem. 2020, 11, 4124-4132.
